# Clinical confocal laser endomicroscopy for imaging of autofluorescence signals of human brain tumors and non-tumor brain

**DOI:** 10.1007/s00432-024-06052-2

**Published:** 2024-12-26

**Authors:** Marlen Reichenbach, Sven Richter, Roberta Galli, Matthias Meinhardt, Katrin Kirsche, Achim Temme, Dimitrios Emmanouilidis, Witold Polanski, Insa Prilop, Dietmar Krex, Stephan B. Sobottka, Tareq A. Juratli, Ilker Y. Eyüpoglu, Ortrud Uckermann

**Affiliations:** 1 Department of Neurosurgery, Faculty of Medicine and University Hospital Carl Gustav Carus, Technische Universität Dresden, Dresden, Germany; 2 Else Kröner Fresenius Center for Digital Health, Faculty of Medicine, Technische Universität Dresden, Dresden, Germany; 3 Medical Physics and Biomedical Engineering, Faculty of Medicine, Technische Universität Dresden, Dresden, Germany; 4 Department of Pathology (Neuropathology), Faculty of Medicine and University Hospital Carl Gustav Carus, Technische Universität Dresden, Dresden, Germany; 5 Division of Medical Biology, Department of Psychiatry and Psychotherapy, Faculty of Medicine and University Hospital Carl Gustav Carus, Technische Universität Dresden, Dresden, Germany

**Keywords:** Autofluorescence, Intraoperative imaging, Label-free, Brain tumor recognition, In situ pathology

## Abstract

**Purpose:**

Analysis of autofluorescence holds promise for brain tumor delineation and diagnosis. Therefore, we investigated the potential of a commercial confocal laser scanning endomicroscopy (CLE) system for clinical imaging of brain tumors.

**Methods:**

A clinical CLE system with fiber probe and 488 nm laser excitation was used to acquire images of tissue autofluorescence. Fresh samples were obtained from routine surgeries (glioblastoma *n* = 6, meningioma *n* = 6, brain metastases *n* = 10, pituitary adenoma *n* = 2, non-tumor from surgery for the treatment of pharmacoresistant epilepsy *n* = 2). Additionally, in situ intraoperative label-free CLE was performed in three cases. The autofluorescence images were visually inspected for feature identification and quantification. For reference, tissue cryosections were prepared and further analyzed by label-free multiphoton microscopy and HE histology.

**Results:**

Label-free CLE enabled the acquisition of autofluorescence images for all cases. Autofluorescent structures were assigned to the cytoplasmic compartment of cells, elastin fibers, psammoma bodies and blood vessels by comparison to references. Sparse punctuated autofluorescence was identified in most images across all cases, while dense punctuated autofluorescence was most frequent in glioblastomas. Autofluorescent cells were observed in higher abundancies in images of non-tumor samples. Diffuse autofluorescence, fibers and round fluorescent structures were predominantly found in tumor tissues.

**Conclusion:**

Label-free CLE imaging through an approved clinical device was able to visualize the characteristic autofluorescence patterns of human brain tumors and non-tumor brain tissue ex vivo and in situ. Therefore, this approach offers the possibility to obtain intraoperative diagnostic information before resection, importantly independent of any kind of marker or label.

## Introduction

Reliable, safe intraoperative tumor analysis is the main challenge in neurosurgery and is promising for improving brain tumor patient therapy. Several techniques, including fluorescence-guided resection, intraoperative magnetic resonance or ultrasound, are clinically used to help neurosurgeons optimizing the extend of resection. However, there is no solution offering in situ brain tumor diagnosis and delineation from the surrounding brain. To overcome this need, new technologies are investigated and analysis of tissue autofluorescence seems to be a promising strategy. Autofluorescence spectra show differences in peak position and overall intensity between brain tumors and normal brain tissue (Pascu et al. 2009), and the spatial pattern of autofluorescence is different for glioblastoma and non-tumor brain tissue (Croce et al. 2003). Multiphoton microscopy revealed that neurons in the CA1 region of the human hippocampus have intense cytoplasmic autofluorescence and that hippocampal layers, sclerotic lesions and blood vessels can be identified both in unstained tissue cryosections and in fresh samples (Uckermann et al. 2017). Different spectral autofluorescence properties and intensities were found in primary and secondary brain tumors compared with non-tumor brain tissues (Galli et al. 2019a). The analysis of autofluorescence patterns allows the identification of brain tumor-specific features, including hypercellularity, nuclear pleomorphism and necrosis, in tissue sections and fresh human brain tumor samples (Chen et al. 2021a; Fürtjes et al. 2023; Galli et al. 2019b; Poulon et al. 2018; Uckermann et al. 2020). Using an experimental endoscope for the acquisition of two-photon autofluorescence, the results were confirmed in a murine orthotopic brain tumor model in vivo (Chen et al. 2021a).

To fully exploit tissue autofluorescence for neurosurgery, safe and reliable in situ label-free imaging needs to be realized in the clinical setting. Confocal laser endomicroscopy (CLE) constitutes a promising candidate technology, as it is already an established intraoperative imaging technology. It combines confocal laser-scanning technology with an endoscopic device and generates high-resolution images of tissue structure at the cellular level, usually using fluorescein as a contrast agent. Commercial systems are approved for neurosurgery, and several studies have shown their safety and potential applications during brain tumor resection (Kakaletri et al. 2022; Restelli et al. 2021; Wagner et al. 2024). It is a fast technology, and CLE systems are able to deliver hundreds of images within minutes of application (Abramov et al. 2023). This underlines the possibility of immediate feedback for the neurosurgeon and makes exploitation in combination with machine learning applications interesting. Proof-of-concept label-free CLE without fluorescent dyes has already been proven for colon and lung cancer (Ellebrecht et al. 2016, 2021), showing that meaningful images can be obtained. Moreover, the technology allowed the assessment of tissue in vivo in an animal model despite of manifold sources of potential disturbances such as moving artifacts, breathing or placement of the probe (Ellebrecht et al. 2019). The autofluorescence of glioma was investigated ex vivo using a preclinical CLE device (Radtke et al. 2024).

All of these previous studies strongly suggest that autofluorescence properties can be exploited for the discrimination of normal brain tissue from brain tumor tissue and hold promise for intraoperative brain tumor diagnosis. However, clinical translation is pending, and the postulated benefit for resection control and intraoperative tumor demarcation remains an open question. Here, we take the first step toward clinical translation and analyze the autofluorescence of fresh human brain tumor samples using a commercial CLE system to assess its potential for intraoperative autofluorescence imaging in neurosurgery.

## Methods

### Patients

Tissue samples for ex vivo label-free CLE imaging were obtained during routine surgery of 25 patients (with one patient undergoing surgery twice: *n* = 26 cases, age range: 27–82 years, median age 63 years; male/female: 6/19). In situ label-free CLE imaging was performed in three cases. This study was performed in line with the principles of the Declaration of Helsinki. Approval was granted by the Ethics Committee of the Technische Universität Dresden (EK-323122008 and BO-EK-104022023). Informed consent was obtained from all individual participants included in the study.

### Samples

Tumor tissue samples were collected from patients with glioblastoma (*n* = 6), meningioma (*n* = 6), brain metastases (*n* = 9), and pituitary adenoma (*n* = 2). Non-tumor brain tissue was obtained from surgical treatment of pharmaco-resistant epilepsy (*n* = 2).

One to four samples were obtained per patient at the discretion of the surgeon (*n* = 35 in total). The samples were transferred into phosphate-buffered saline (PBS) immediately after surgical removal, and label-free CLE imaging was performed within 2 h.

The samples were subsequently subjected to formalin fixation and cryoprotection in 10% and 30% sucrose solutions before cryopreservation at -80 °C. Tissue cryosections were prepared and stained with hematoxylin and eosin (HE) for reference.

### CLE device and image acquisition

A Convivo CLE device (Zeiss, Oberkochen, Germany) was used for ex vivo and in situ imaging. It consists of a scanner unit with a handheld probe, which couples with a user station. A touchscreen allows for intraoperative setting adjustments and displays the acquired images. The system scans a field of view of 267 × 475 μm and provides images with a size of 1920 × 1080 pixels. Excitation was provided by a laser source at a wavelength of 488 nm, and either the green bandpass (BP, 518–573 nm) or longpass (LP, > 515 nm) emission filter was used to acquire tissue autofluorescence. The laser power was set to 100%, with a gain of 2400–2700, and the autobrightness setting was switched on.

To reduce motion artifacts during ex vivo imaging, the Convivo probe was fixed in a mounting, and the sample was placed in a Petri dish customized with a nylon mesh. In situ label-free CLE images (total: 338, range of 100–132) were acquired at different positions in the tumor or hippocampus as assessed by the neurosurgeon.

### Image and data analysis

Images containing obvious motion artifacts, showing an identical or very similar field of view, and images without any discernible signal were excluded.

Image analysis was performed via Fiji (Schindelin et al. 2012) The signal‒to-noise ratio (SNR) was analyzed in matched BP and LP images of the same field of view. Gray values were extracted along a line crossing a fluorescent object, and values were allocated to originate from either the background or the signal. The difference in the mean gray values for the signal and background was divided by the standard deviation of the background signal SNR = ∆µ/σ.

Nine features were identified in label-free CLE images of brain tumors: (1) Sparse punctuated autofluorescence signals (diameters of 1–3 μm, < 50 signals/field of view); (2) Dense punctuated autofluorescence signals (diameters of 1–3 μm, ≥ 50 signals/field of view); (3) Sparse small cells with fluorescent cytoplasm (< 5 cells /field of view, < 25 μm in diameter); (4) Dense small cells with fluorescent cytoplasm (≥ 5 cells /field of view, < 25 μm in diameter); (5) Sparse large cells with fluorescent cytoplasm (< 5 cells /field of view, ≥ 25 μm in diameter); (6) Dense large cells with fluorescent cytoplasm (≥ 5 cells /field of view, ≥ 25 μm in diameter); (7) Diffuse autofluorescence; (8) Fibers; (9) Round homogenous structures. The images were manually inspected for the presence of these features by a person blinded to the type of tissue/brain tumor. The analysis was performed using constant image size, monitor brightness and contrast settings on unprocessed images.

Glyph plots were calculated using the function glyphplot option ‘face’ in MATLAB 2023a (The MathWorks, Inc). The features were transformed into facial features in the following manner: size of face: sparse punctuate autofluorescence, forehead/jaw relative arc length: diffuse autofluorescence, shape of forehead: fibers, shape of jaw: dense punctuate autofluorescence, angle of eyes: round structures, direction of pupils: sparse small cells, length of nose: dense large cells, shape of mouth: dense small cells, mouth arc length sparse large cells. All other facial features were displayed at their default value.

Prism 10 (GraphPad Software, Inc) was used to create graphs and perform statistical analysis of the data. The type of test is specified in the manuscript text. Affinity Photo 2 (Serif Europe Ltd) was used to process images for display purposes applying noise reduction, sharpening filters and brightness/contrast enhancement.

### Label-free multiphoton microscopy

For reference, label-free multiphoton microscopy (MPM) was performed on tissue cryosections. The sections were thawed, rehydrated with PBS and coverslipped. Autofluorescence (AF), coherent anti Stokes Raman scattering (CARS), and second harmonic generation (SHG) were acquired as described previously (Galli et al. 2019b). The multiphoton microscope is comprised of an LSM 7 laser scanning module coupled to an Axio Examiner Z.1 upright microscope, equipped with a W Plan Apochromat 20 × /1.0 objective (all Carl Zeiss Microscopy GmbH, Jena, Germany). Two erbium fiber lasers (Femto Fiber pro NIR at 781 nm and TNIR at 1005 nm, Toptica Photonics AG, Gräfelfing, Germany) with a pulse length of approximately 1 ps were used. All signals were simultaneously excited and detected with the appropriate optical filtering. The autofluorescence signal was acquired using a bandpass filter 500–550 nm. For CARS (depicting symmetric stretching vibration of methylene groups at 2850 cm⁻¹) and SHG (showing mainly collagen) bandpass filters 626–654 nm and 372–408 nm were used, respectively. After MPM, the imaged tissue section was HE stained to enable direct one-to-one matching of the tissue structures.

## Results

**Fig. 1:**
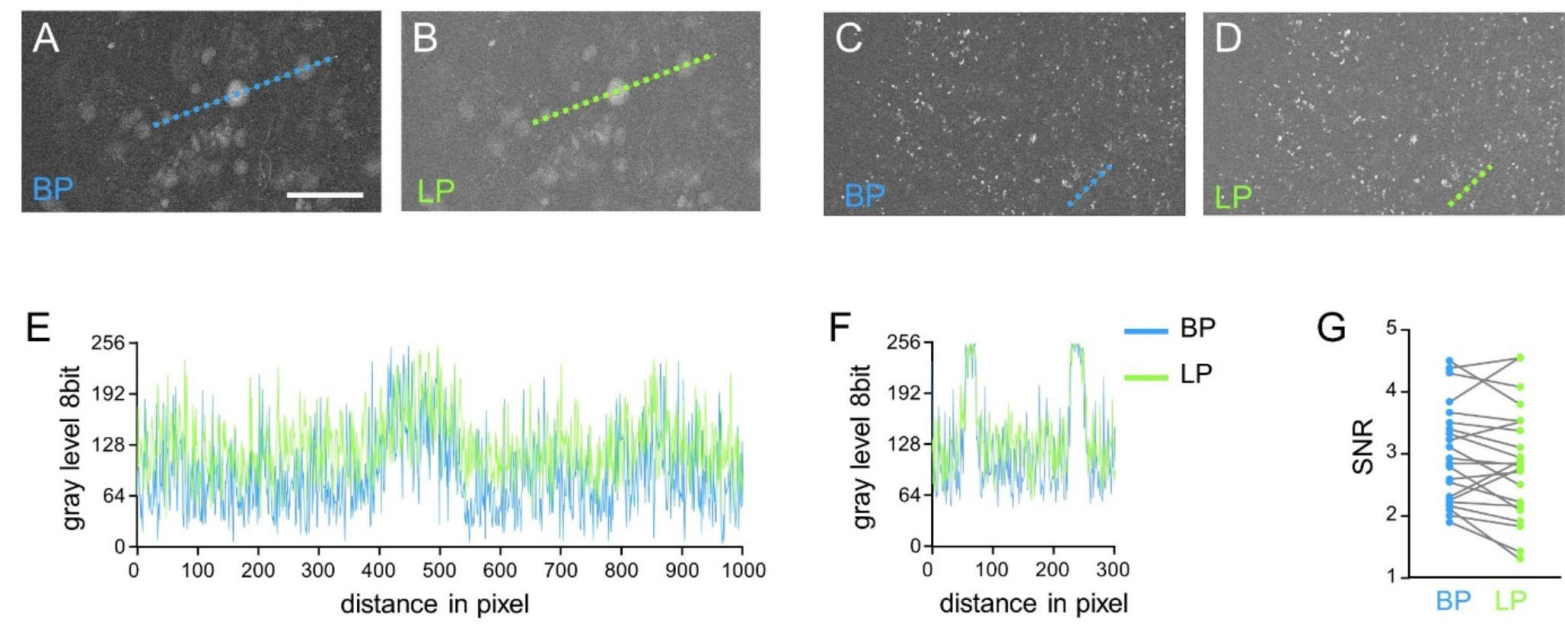
Comparison of detection filters for label-free CLE imaging. **A/B**: Autofluorescence images of a fibrous meningioma of WHO CNS grade 1. **C/D**: Autofluorescence images of a glioblastoma. **E**: Plots of gray levels along the lines indicated in A and B. **F**: Plots of gray levels along the lines indicated in D and E. **G**: Signal‒noise ratios (SNR) of paired autofluorescence images (*n* = 22 cases; for 4 cases, no matched BP/LP images were available). BP: bandpass filter; LP: longpass filter; scale bar indicates 100 μm

Label-free CLE enabled the acquisition of images of tissue autofluorescence of all the samples investigated. First, we investigated which of the system’s detection filters is more effective for autofluorescence imaging. Therefore, images were acquired using the autobrightness function and either the bandpass (BP 518–573 nm) or the longpass (LP > 515 nm) filter. Examples of paired label-free CLE images of the same field of view acquired with either filter are shown for meningioma (Fig. 1A, B) and glioblastoma (Fig. 1C, D). No differences in the pattern of autofluorescence, size or distribution of objects were found upon visual inspection. The intensity plots indicate lower background levels for the BP filter in comparison to the LP filter (Fig. 1E, F). The investigation of paired images revealed no difference in the signal-to-noise ratio (SNR, Fig. 1G, *n* = 22, median SNR BP: 2.89, median SNR LP: 2.84, *P* = 0.1129, Wilcoxon test). However, the darker background in BP images gives the visual impression that autofluorescent structures are easier to see. Therefore, the BP filter was selected for acquisition of label-free CLE images in this study.

**Fig. 2:**
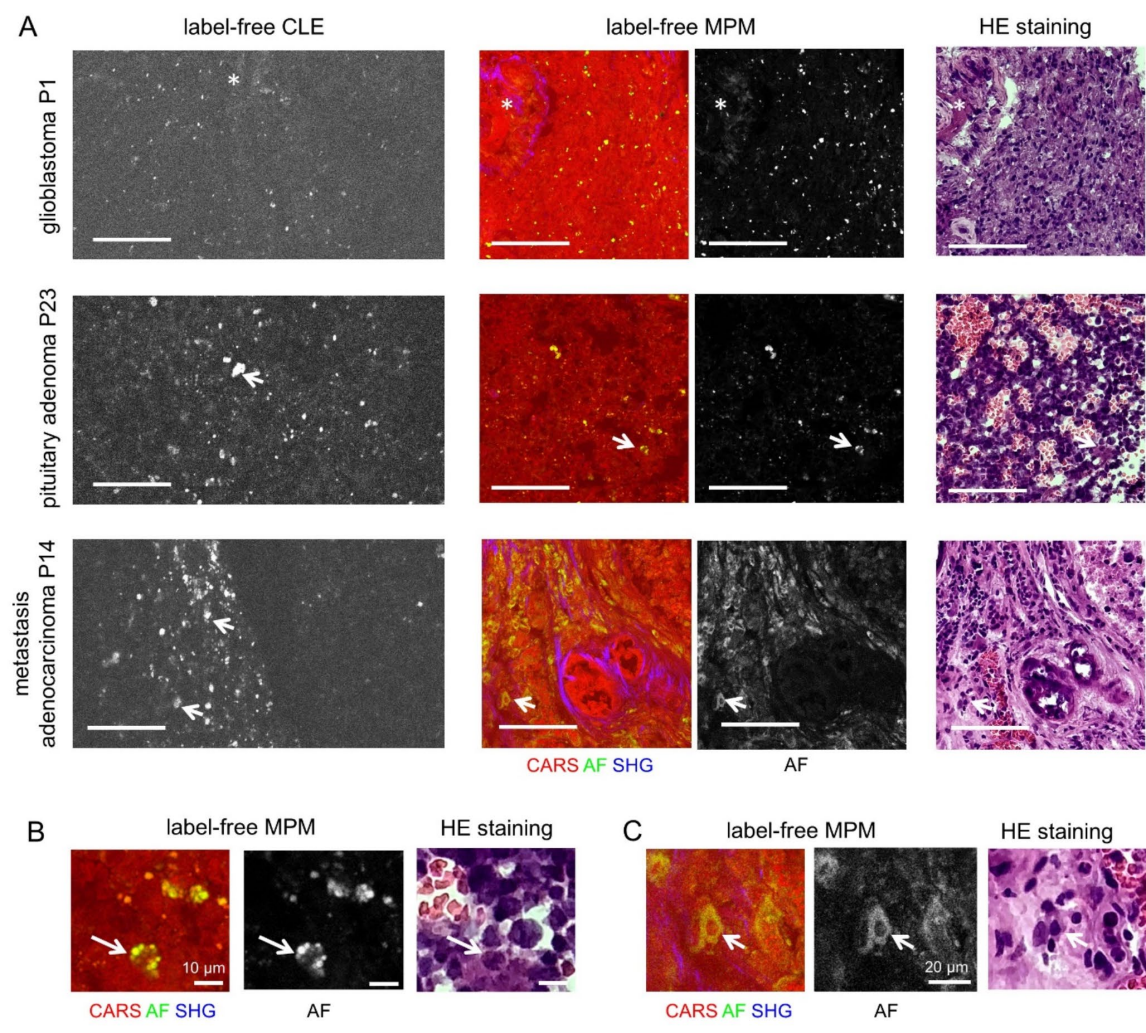
Label-free CLE of brain tumors ex vivo in reference to label-free MPM and HE-matched histology. **A**: Examples of a case of glioblastoma, pituitary adenoma and brain metastasis of adenocarcinoma, scale bars 100 μm. **B**: Magnification of the MPM images and HE reference staining of the pituitary adenoma. **C**: Magnification of the MPM images and HE reference staining of the metastasis of adenocarcinoma, arrows indicate autofluorescent cells, asterisks indicate blood vessels

A total of 6847 autofluorescence images were acquired from fresh specimens using the clinical CLE system, (range 102–923, in median 220 images per case), with 1114 of these subjected to subsequent analysis (range: 17–115, in median 37 images per case). Figure 2A shows examples of label-free CLE images of different types of brain tumors ex vivo (left). For reference, label-free multiphoton microscopy (MPM) was performed on unstained tissue sections prepared from those samples (middle), which revealed that the autofluorescence signal (AF, green) was in accordance with the tissue autofluorescence observed in the label-free CLE images. The label-free CLE image of the glioblastoma shows a punctuate pattern of small autofluorescent structures (~ 1–3 μm in diameter). Those are likewise present in reference multiphoton imaging (AF channel) but cannot be assigned to a certain cell or tissue structure upon comparison with HE staining matched to the position of the MPM. Weak autofluorescence was found to be related to the presence of blood vessels (asterisk). The label-free CLE image of the pituitary adenoma shows likewise a punctuate pattern of autofluorescence. Occasionally, agglomerates of strongly fluorescent structures are found (arrows), which were located in the cytoplasm of cells based on shape, size and direct one-to-one referencing between high magnification label-free MPM images and HE staining (Fig. 2B). The direct comparison further illustrates that erythrocytes are not visible in autofluorescence images. The label-free CLE image of a brain metastasis of an adenocarcinoma appears in two parts. On the left side, there is a strong, heterogeneous autofluorescence pattern, while only a few punctuate structures are visible on the right side. As mentioned, this is in concordance with the label-free MPM images. The areas without autofluorescence can be assigned to tumor cells and collagen-rich epithelial tissue formations. Many cells display a cytoplasmic autofluorescence (see Fig. 2C for high magnification), however it appears weaker and more diffuse than in the cells of the pituitary adenoma. The comparison with the HE-staining suggests, that the fluorescent cells might represent inflammatory macrophages and/or fibroblasts of the tumor stroma.

**Fig. 3:**
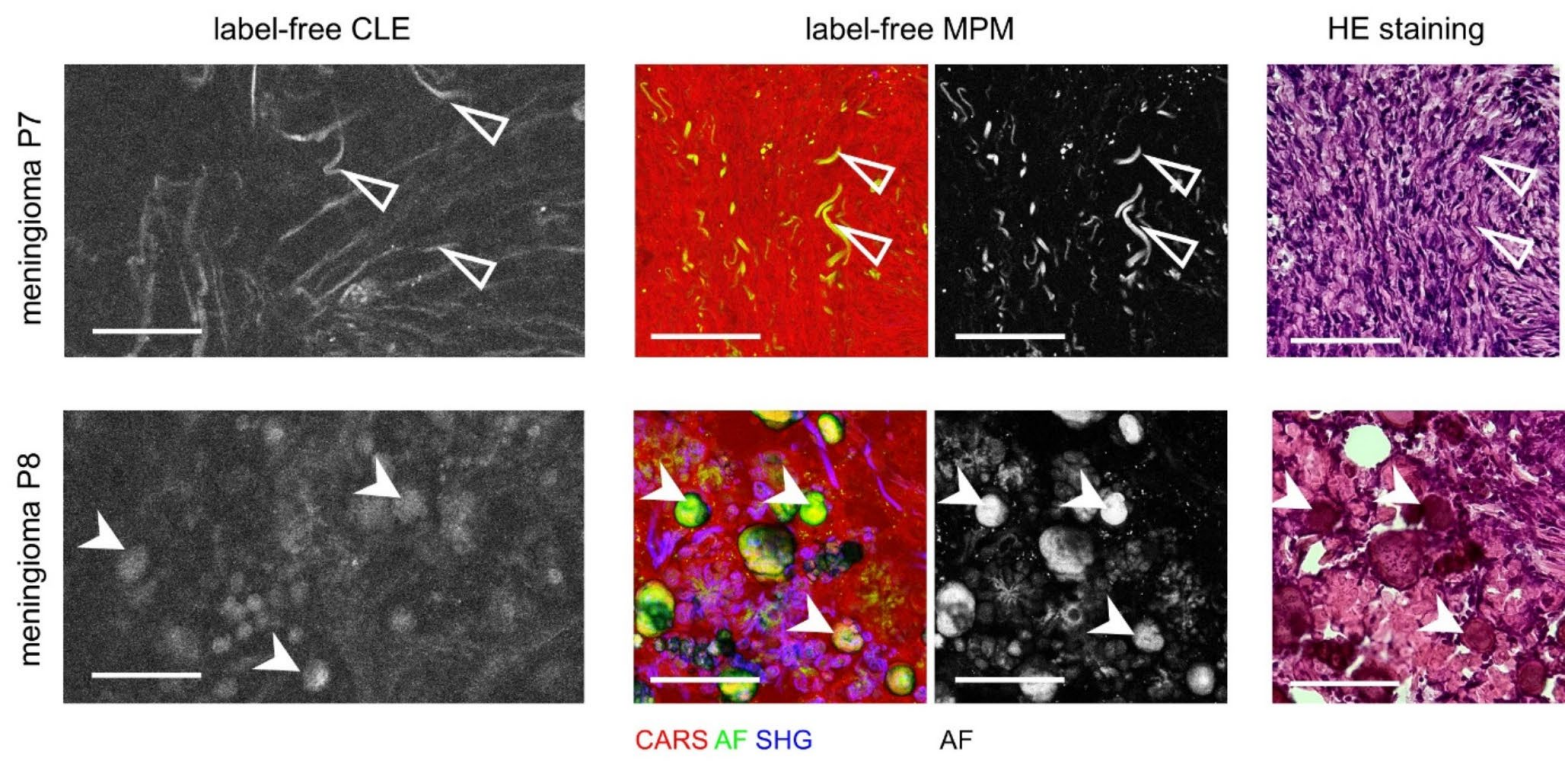
Label-free CLE of meningioma ex vivo in reference to label-free MPM and HE-matched histology. The examples show two cases of meningiomas. Triangles indicate fibers, arrowheads indicate psammoma bodies, scale bars 100 μm

Figure 3 shows two cases of meningiomas and illustrates that the autofluorescence signals are largely variable and reveal different tissue properties. In the first example (meningioma P7), thin linear autofluorescent structures are visible (open arrowheads). These are likewise visible in reference label-free MPM and presumably represent elastin fibers; fibrillar collagen would have been identified by SHG signals in multiphoton images (Chen et al. 2012). The label-free CLE image of the second example (meningioma P8) is dominated by round structures with a diameter of 20–60 μm which display weak autofluorescence (filled arrowheads). Similar structures are found by MPM. Reference HE staining identifies those structures as psammoma bodies. Autofluorescence signals of psammoma bodies and fibers have been observed in fresh meningioma samples ex vivo (Fürtjes et al. 2023).

**Fig. 4:**
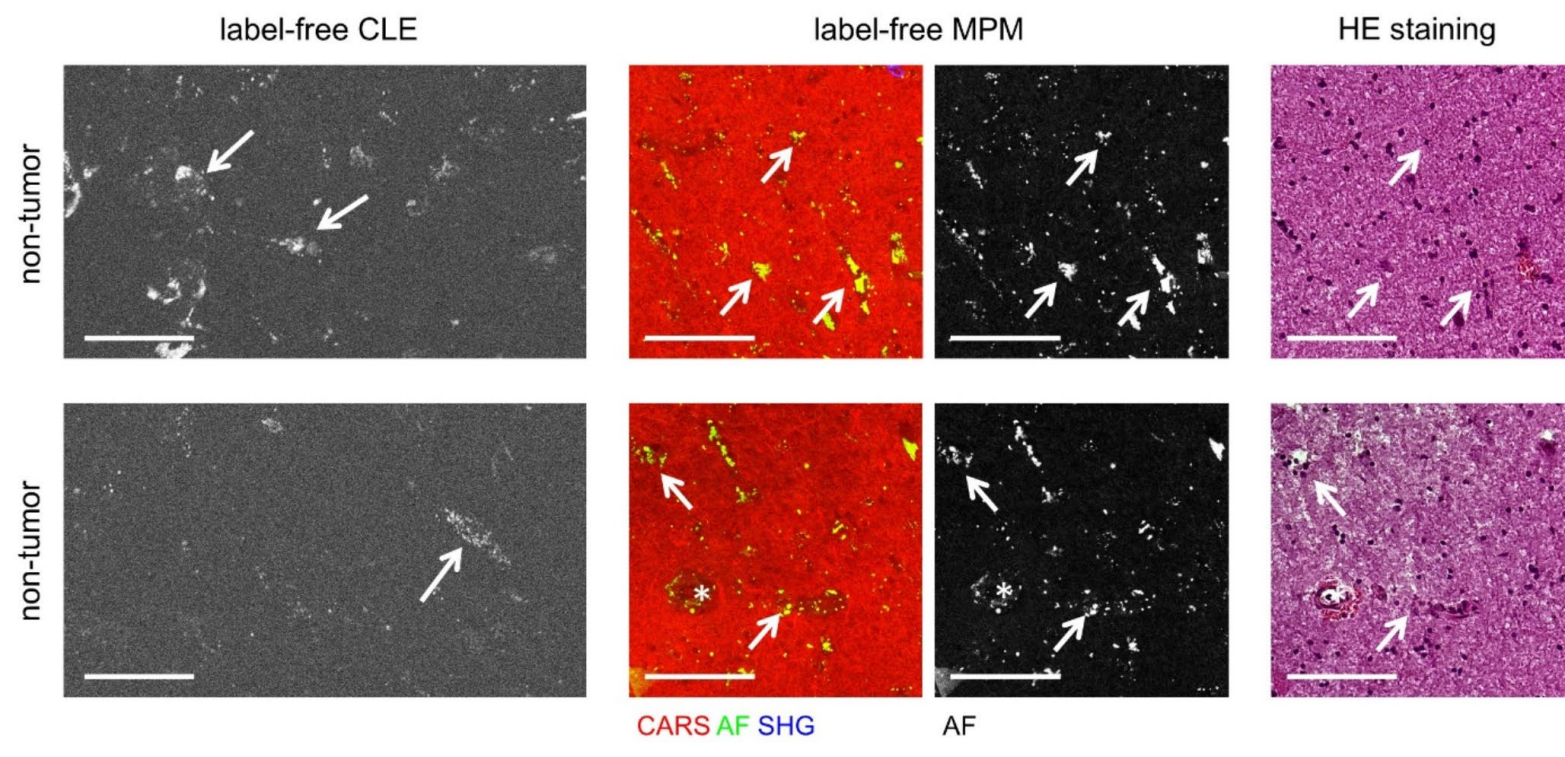
Label-free CLE of non-tumor brain ex vivo in reference to label-free MPM and HE histology. Arrows indicate autofluorescent cells, asterisks indicate blood vessels. Scale bars 100 μm

Label-free CLE images of non-tumor brain tissue from epilepsy surgery show that one part of the autofluorescence signal originates from small punctuate structures. Additionally, larger agglomerates of fluorescent structures were found and identified as the cytoplasmic compartment of cells in comparison with reference HE (Fig. 4, arrows). Moreover, blood vessels might also contribute to the autofluorescence signal in non-tumor tissue, as similarly observed in brain tumors (Figs. 2 and 4, asterisks).

**Fig. 5:**
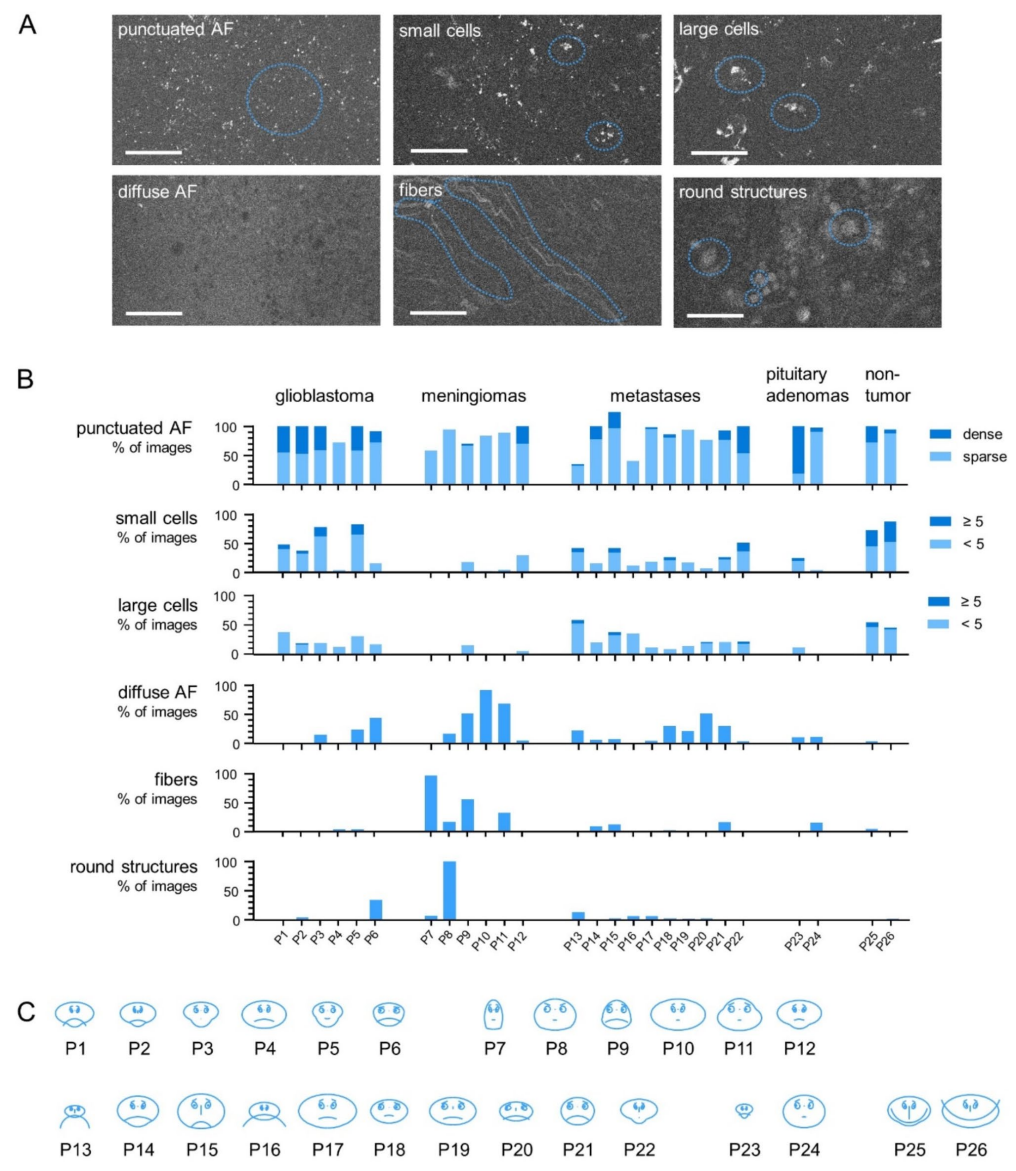
Abundance of autofluorescent features in brain tumors and non-tumor brain. **A**: Examples of label-free CLE images showing the features indicated by the corresponding labels, selected examples are additionally highlighted by blue dotted lines. Scale bar 100 μm **B**: All interpretable label-free CLE images obtained from each case (P1-P26) were inspected for presence of autofluorescent features. The bars show the percentage of the images in which the respective feature was detected. **C**: Glyph plot of the abundance of autofluorescence features in CLE images for all cases (Chernoff face; size of face: sparse punctuate autofluorescence, forehead/jaw relative arc length: diffuse autofluorescence, shape of forehead: fibers, shape of jaw: dense punctuate autofluorescence, angle of eyes: round structures, direction of pupils: sparse small cells, length of nose: dense large cells, shape of mouth: dense small cells, mouth arc length sparse large cells)

Based on inspection of label-free CLE images of different brain tumor types and of non-tumor brain, the following features were identified as being frequently present and potentially meaningful (Fig. 5A): Punctuate autofluorescence signals were either restricted to some areas or present within the entire field of view. Those small fluorescent structures typically had diameters of 1–3 μm and were either loosely distributed or more densely packed. Cells of various sizes were identified by presence of strong granular or more diffuse autofluorescence in the cytoplasmic compartment. In some cases, diffuse autofluorescence signal within the field of view with negative contrast of round structures was observed. Fiber-like structures presented with an autofluorescence signal and exhibited a variety of shapes, including straight and curved lines and undulating shapes. Round structures displayed a homogenous weak autofluorescence. They had diameters between 10 and 60 μm and typically occurred in clusters. Several features were often present in the same image.

Subsequently, the presence of these features was evaluated in all samples to determine their potential for tumor identification. Figure 5B shows the presence of those features for the different types of tumors and for non-tumor tissue. While statistical analysis is not reasonable on low sample numbers because of the high intra and inter tumor variability, the graph nevertheless gives an idea of the typical autofluorescence features of the respective tumor type and in non-tumor brain. Punctuated autofluorescence is found in most images across all cases, and is more dense in glioblastomas. Moreover, one of the two cases of pituitary adenoma showed dense punctuated autofluorescence in 80% of images. Small cells were found in most tumor cases, although the proportion of CLE images with cells varied greatly (in median glioblastoma: 43%, meningiomas: 4% metastases: 23%, pituitary adenoma: 15%). In the non-tumor samples, small cells were more frequently detected (in 73% and 88% of images, respectively) and observed in higher densities. The prevalence of large cells was comparable to that of small cells, albeit at a lower frequency. Diffuse autofluorescence was found in at least some label-free CLE images of all tumor types, with highest abundance in meningiomas. Fibers were present in meningiomas (4/6 cases), metastases (3/10 cases) and pituitary adenomas (1/2 cases), with the highest prevalence in meningiomas. Round fluorescent structures were occasionally found in images of glioblastomas, meningiomas and metastases, and with higher rate of occurance in one case of glioblastoma (32% of images) and one meningioma (94% of images).

Nevertheless, it remains challenging to draw conclusions from the autofluorescence features of a specific case and to conduct a comprehensive comparison between cases when the data are presented in a purely numerical format. Therefore, an alternative approach for visualization of multivariate data was tested and we transformed the data into a glyph plot in the form of a human face (Chernoff 1973). The nine features analyzed in the CLE images were converted into facial features (Fig. 5C). As humans are innately adept at recognizing faces and are sensitive to even subtle changes, this type of graph facilitates the identification of cases with similar features, for example a case of glioblastoma (P3) and a case of metastases (P22). In this representation of the data, non-tumor cases (P25 and P26) can be distinguished from the tumor cases by shape and size of the mouth, the length of the nose and position of the pupils, all of which are calculated based on the abundance of cells. This further illustrates, that the presence of autofluorescent cells is the main feature of non-tumor brain tissue that is disrupted by tumor growth. Moreover, the glyph corresponding to the case of glioblastoma (P5), which showed a similar abundance of autofluorescence cells as the non-tumor sample but additional tumor-related features, looks markedly different.

As distinct cell and tissue related autofluorescent features were identified in ex vivo label-free CLE images, the technology qualified as interesting intraoperative tool. Therefore, label-free CLE was performed in situ during resection in three neurosurgical cases as proof-of-concept. The handling of the probe and the acquisition of the images were seamless and were carried out without problems. However, images were often affected by artifacts, as also described for fluorescein-CLE (Abramov et al. 2023), including motion artifacts, large out-of-focus areas and contamination with blood. Of the 338 label-free CLE images acquired in situ, 53 images (16%) exhibited no AF signal, 141 images (42%) contained motion artifacts, 73 images (22%) showed tissue related structures and were used for feature quantification while 71 images visualized similar field-of views and were excluded from analysis. Fluorescein-CLE has been described to deliver a similar percentage of images with identifiable histological features (Abramov et al. 2023).

**Fig. 6:**
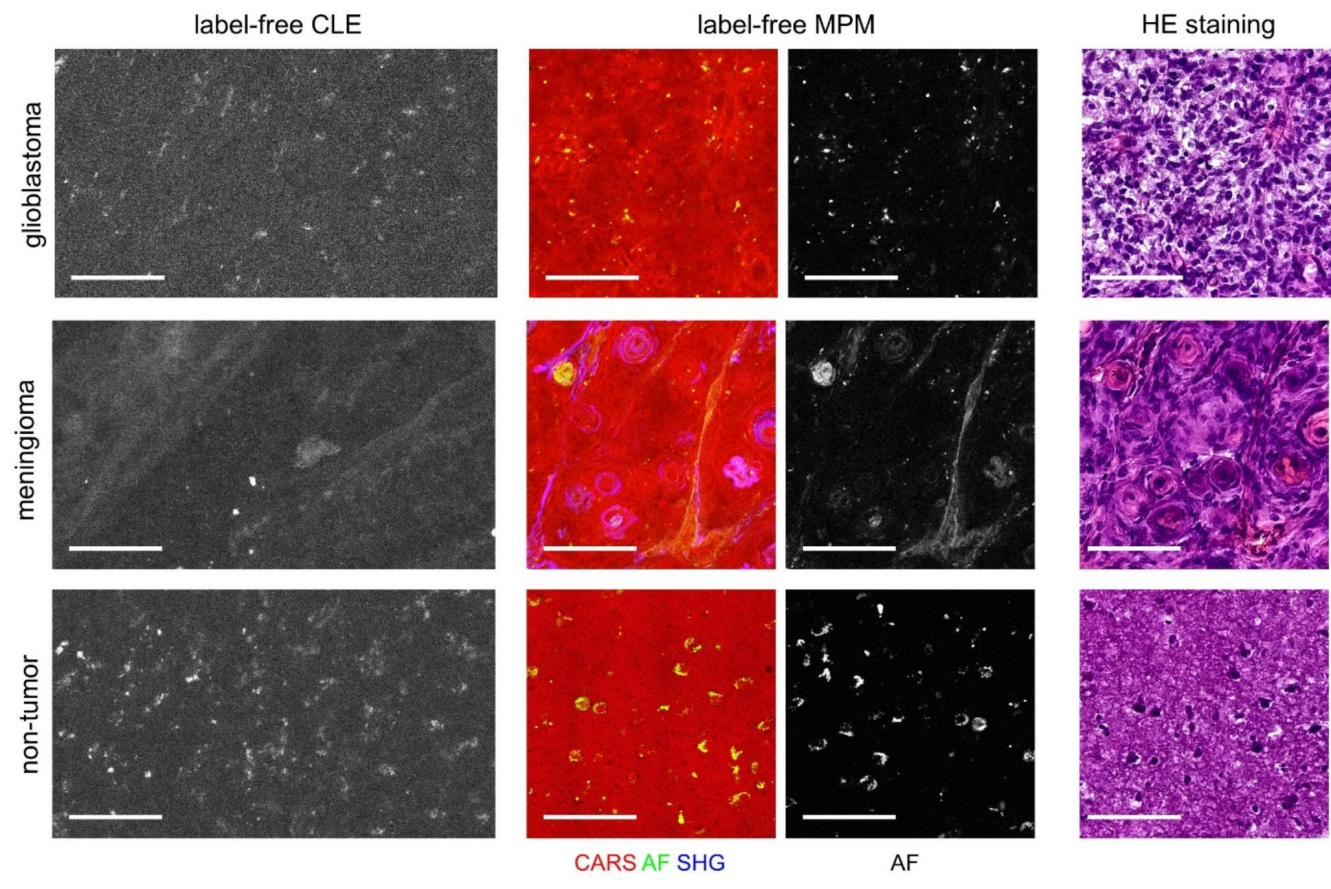
Proof-of-concept intraoperative label-free CLE in situ. Examples of glioblastoma, meningioma and non-tumor tissue are shown in reference to label-free MPM and HE histology of the tissue sample resected after in situ label-free CLE imaging. Scale bars 100 μm

Examples of autofluorescence images acquired in situ during neurosurgical resections are shown in Fig. 6 and demonstrate that the features that were found ex vivo (compare Figs. 2, 3, 4 and 5) can be also identified during in situ imaging. Punctuate autofluorescence is visible in the label-free CLE images of glioblastoma, meningioma and non-tumor. The case of meningioma was further characterized by the presence of fiber-like as well as of round fluorescent structures, presumably elastin fibers and psammoma bodies. Small cells were visible in images of glioblastoma and non-tumor brain tissue, confirmed by retrospective ex vivo MPM and HE histology.

## Discussion

The autofluorescence or endogenous fluorescence of cells and tissue is often seen as disturbing background or pitfall when analyzing fluorescence staining or the expression of fluorescent proteins. It is considered a disturbing signal during fluorescence-guided resection in neurosurgery (Black et al. 2021). However, the acquisition of autofluorescence visualizes several tissue structures and the most important endogenous fluorophores are pyridinic (NAD(P)H) and flavin coenzymes, lipofuscins, advanced glycation end products, and collagen and elastin (Monici 2005). Several studies showed the potential of autofluorescence signals of brain tumors for tissue classification and diagnosis as well as the recognition of non-tumor brain (Chen et al. 2021b; Fürtjes et al. 2023; Galli et al. 2019a; Radtke et al. 2024; Uckermann et al. 2020). Moreover, analysis of autofluorescence in the time domain expands assessing the metabolic state of brain tumor cells (Alfonso-García et al. 2022; Reichert et al. 2023; Yuzhakova et al. 2023).

The results of our study suggest that label-free CLE can provide meaningful images of tissue autofluorescence of human brain tumors and non-tumor brain using a system approved as medical product for neurosurgery. The features found in this study are similar to autofluorescence structures identified by ex vivo microscopy in brain tumors (Galli et al. 2019b) and, therefore, discrimination of tumor type and delineation of brain tumors might be likewise possible using label-free CLE images. Our data confirm that a high number of autofluorescent cells and the additional absence of diffuse autofluorescence, fibers and psammoma bodies is an indicator for non-tumor tissue.

In situ imaging poses a number of inherent challenges: movement artifacts and blood contaminations have to be identified or, even better, avoided. Moreover, as the technique is confocal, finding the correct imaging plane in z-direction might be an issue when working in the intraoperative setting (is there no signal or is the acquisition plane wrong?) especially when working with low intensity autofluorescence. This illustrates the need for future development of clinical protocols.

The label-free CLE images are characterized by strong noise. This might result from the detector in combination with the high laser gain and brightness settings that were used. These are necessary as autofluorescence signals are less intense than fluorescein fluorescence for which the system is designed. Nevertheless, even without image processing, autofluorescent structures were visualized and identified as cells, fibers and psammoma bodies and punctuate and diffuse autofluorescence was observed. This is of utmost importance as those non-processed images are displayed on the screen of the instrument in real-time and allow the surgeon to inspect and evaluate suspicious tissue. The optimal visibility of tissue autofluorescence was achieved in a darkened operating room, which was comparable to the illumination conditions utilized for fluorescence-guided resection with 5-ALA. Hence, advanced image processing tools and filters might be suitable to denoise images (Meiniel et al. 2018).

Extraction of tissue type and diagnosis from microscopy images is usually performed by image analysis, including enhancement, automated features extraction and classification (Kumar et al. 2015). In this context, deep learning is considered as very promising (Hewitt et al. 2023; Hollon et al. 2023). In our study, the image analysis was limited to visual inspection of label-free CLE images and manual assessment of features. This strategy mimics the neurosurgical application for in situ tissue analysis envisioned in the near future. Further developments of CLE systems might include image segmentation, feature recognition algorithms and/or classifiers to help the surgeon with image interpretation.

Fluorescein-CLE has been employed for intraoperative imaging of brain tumors. However, it relies on uptake of fluorescein by the tumor through an impaired blood-brain-barrier (Belykh et al. 2020) and, therefore, faces intrinsic limitations for visualization of non-enhancing tumors and non-tumor brain pathologies as well as for detection of the tumor border in case of infiltrations (Xu et al. 2024). Label-free CLE as reported in this study might be able to overcome these limitations. Moreover, autofluorescence offers the possibility to visualize additional tumor properties that are not accessible by fluorescein CLE. For instance, recent research has indicated that autofluorescence signals can be exploited for characterization of the tumor microenvironment (You et al. 2021). Moreover, activated microglia/macrophages display intense autofluorescence after CNS injury (Uckermann et al. 2015). These findings suggest that the components of the tumor microenvironment might significantly contribute to the autofluorescence signals of brain pathologies might be assessable by label-free CLE.

The advantage of autofluorescence imaging over other existing or research technologies is the simplicity of the approach: no contrast agent is needed, saving patients from adverse effects and the surgical team from additional organizational issues. Moreover, any issues related to timing of administration, dye uptake, distribution and excretion are non-existing, and reliable acquisition of autofluorescence signals is possible at any time point of the surgery. Our study clearly shows that autofluorescence signals of brain tumors and non-tumor brain tissue can be acquired with a clinical CLE system and that meaningful images can be obtained in situ before tissue removal. This intraoperative assessment of cell and tissue microstructure without the need to apply any contrast agents or fluorophores opens up completely new possibilities of brain tumor research, diagnosis and treatment.

## Data Availability

The data of this study are not openly available due to ethical reasons and are available from the corresponding author upon reasonable request under a data use agreement.
